# Two new species of *Stenandra* Lameere, 1912 (Coleoptera, Cerambycidae, Parandrinae) from the Oriental Region

**DOI:** 10.3897/zookeys.103.1404

**Published:** 2011-06-10

**Authors:** Ziro Komiya, Antonio Santos-Silva

**Affiliations:** 1 3-2-12, Shimouma, Setagaya-ku, Tokyo, 154-0002, Japan; 2 Museu de Zoologia, Universidade de São Paulo. Caixa Postal 42594, 04299-970 São Paulo, São Paulo, Brazil

**Keywords:** new species, Sulawesi, Vietnam

## Abstract

Two new species of the genus *Stenandra* Lameere, 1912 are described: *Stenandra saitoae* from Sulawesi of Indonesia and *Stenandra asiatica* from Vietnam. A key to the species of the *Stenandra* is given.

## Introduction

Lameere (1912) erected *Stenandra* as a subgenus of *Parandra* Latreille, 1802, for *Parandra kolbei* Lameere, 1903 (Figs 5, 6) from the Ethiopian Region. Quentin and Villiers (1972) considered *Stenandra* as a genus distinct from *Parandra*, and described the second species: *Stenandra vadoni* (Fig. 4) from Madagascar.

Recently, Santos-Silva et al. (2010) published a revision of the Parandrinae of the Hawaiian, Australasian, Oriental, and Japanese regions. In that paper, the authors misidentified a specimen collected in Vietnam as *Stenandra kolbei*, incorrectly believing that the species was introduced into this country with imported wood.

*Stenandra* differs from all other genera of Parandrini, except *Neandra* Lameere, 1902, by the absence of a paronychium. From *Neandra*, an exclusively North American genus, it differs by the presence of setae on the elytra (glabrous in *Neandra*).

In the redescription of the genus in Santos-Silva et al. (2010) wrote: “Antennae (Fig. 215) surpassing base of elytra; ventral sensorial area of antennomeres III-XI visible from side, divided by strong carina; dorsal sensorial area of antennomere XI large, deep, well delimited”. However, nearly all information agrees with the species from the Oriental Region, but not with the species from the Ethiopian Region, except length of antenna. Thus, it is necessary to amend as: ventral sensorial area of antennomeres III-XI visible or not in lateral view, divided by distinct carina; dorsal sensorial area of antennomere XI, deep and well delimited.

The presence of *Stenandra* in Vietnam and Sulawesi suggests that the genus probably occurs in other areas of the Oriental zoogeographical region, possibly in Borneo. However, specimens of the genus from Africa are poorly represented in collections. The Muséum National d’Histoire Naturelle (Paris, France) apparently has the largest collection of *Stenandra* and researchers that have collected extensively in Africa in the last several years, such as Norbert Delahaye and Karl Adlbauer, have got very few specimens. Outside of Africa and Madagascar, the two females studied here are all that are so far. According to Tavakilian (pers. comm.) the males of the genus seem to be particularly rare but we are looking forward to the first male, as well as additional females, from the Oriental region.

In this paper, we describe two new species of *Stenandra* and provide a key to the species in the genus.

The collection acronyms used in the text are as follows:

**MZSP**	Museu de Zoologia, Universidade de São Paulo, São Paulo, Brazil;

**NSMT**	National Museum of Nature and Science, Tokyo, Japan.

## Key to the species of Stenandra

**Table:** 

1	Distance between anterior apex of upper eye lobe and posterior end of antennal insertion equal to the length of scape (Fig. 1). Madagascar	*Stenandra vadoni* Quentin & Villiers, 1972
–	Distance between anterior apex of upper eye lobe and posterior end of antennal insertion much shorter than the length of scape (Fig. 2)	2
2 (1)	Carina of ventral sensorial area of antennomeres III-XI not visible in lateral view (Figs 7, 8) (sometimes slightly visible at apical antennomeres). Intertropical Africa	*Stenandra kolbei* (Lameere, 1903)
–	Carina of ventral sensorial area of antennomeres III-XI distinctly visible in lateral view (primarily the apical antennomeres) (Figs 11, 14)	3
3 (2)	Dorsal sensorial area of antennomere XI shorter than one-sixth of total length (Fig. 10); elytral suture divergent apically. Indonesia (Sulawesi)	*Stenandra saitoae* sp. n.
–	Dorsal sensorial area of antennomere XI longer than one-fourth of total length (Fig. 13); elytral suture not divergent apically. Vietnam	*Stenandra asiatica* sp. n.

**Figures 1–8.**
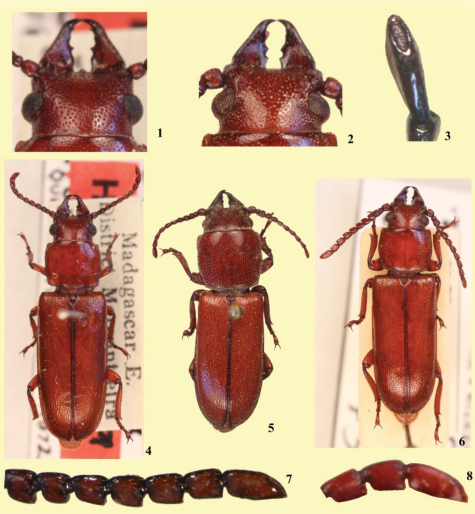
*Stenandra vadoni* Quentin & Villiers, 1972, holotype male **1** Head, dorsal view **4** Habitus **8** Antennomeres, lateral view. *Stenandra kolbei* (Lameere, 1903) **2** Head, dorsal view, female **3** Antennomere XI, dorsal view **5** Male, habitus **6** Female, habitus **7** Antennomeres, lateral view, female. Photos from Gérard Tavakilian (MNHN), except figure 3 (from Kiyoshi Matsuda, Japan – specimen from MNHN).

## Taxonomy

### Stenandra saitoae sp. n.

urn:lsid:zoobank.org:act:FDDB9174-8FB0-4B5F-8DE9-45C1E8CE97BD

http://species-id.net/wiki/Stenandra_saitoae

Figs 9–11 

#### Etymology.

Dedicated to Dr. Akiko Saito of the Natural History Museum and Institute, Chiba, who collected the holotype.

#### Diagnosis.

**Figures 9–14.**
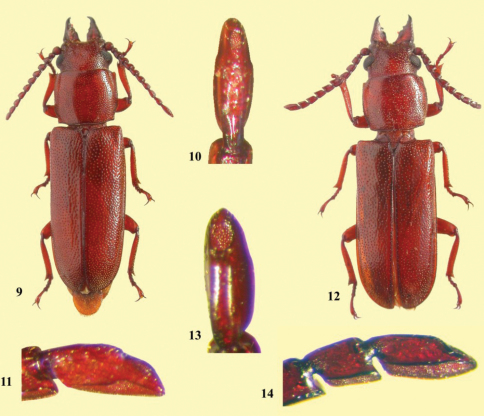
*Stenandra saitoae* sp. n., holotype female **9** Habitus **10** Antennomere XI, dorsal view **11** Antennomere XI, lateral view. *Stenandra asiatica* sp. n., holotype female **12** Habitus **13** Antennomere XI, dorsal view **14** Antennomere XI, lateral view.

 *Stenandra saitoae* sp. n. (Fig. 9) differs from *Stenandra vadoni* Quentin & Villiers, 1972 (Fig. 4), mainly by anterior upper eye edge (Fig. 9) placed close to base of antennae (distant from base of antennae in *Stenandra vadoni* – Fig. 1). From *Stenandra kolbei* (Lameere, 1903) (Fig. 6) it differs by the carina of ventral sensorial area of antennomeres III-XI distinctly visible in lateral view (Fig. 11), dorsal sensorial area of antennomere XI small (Fig. 10), antennomeres enlarged towards inferior edge (Fig. 10), and suture of the elytra apically divergent. In *Stenandra kolbei*, the carina of ventral sensorial area of antennomeres III-XI is not or very slightly visible in lateral view (Fig. 7), the dorsal sensorial area of antennomere XI is large (Fig. 3), antennomeres not distinctly enlarged towards inferior edge (Fig. 3), and the elytral suture is not divergent at the apex. From *Stenandra asiatica* sp. n. it differs mainly by the small sensorial area of antennomere XI (Fig. 10), the more sparse elytral punctation, and by the elytral suture divergent apically.

Female: Integument dark-brown; parts of the mandibles, margins of the pronotum, elytral suture, pro- and mesosternal process, trochanters, extreme ventral apex of tibiae blackish; elytra laterally and apically darker than the remaining surface.

Dorsal area of head coarsely punctate; punctures sparser at central area, coarser, more abundant and confluent at side; punctures between upper eye lobes with short, apically spatulate setae; punctures between posterior eye margin and margin of pronotum with very short, thick setae. Area behind eyes coarsely, confluently punctate near apices of upper eye lobes, sparsely near lower eye lobes; nearly all punctures with single, very small, thick seta. Clypeus coarsely, densely punctate, except at smooth central area; punctures with short, spatulate setae. Labrum smooth, glabrous laterally; coarsely punctuate; with short, spatulate setae around central projection. Upper eye lobe not notably separated from base of antenna. Submentum coarse; densely punctate near anterior margin (confluently centrally), sparsely and coarsely near gena; each puncture with a small, spatulated seta; anterior edge slightly elevated throughout. Mentum with dense, spatulate setae, somewhat longer laterally. Mandibles punctate; external punctures coarse, deep, dense and accompanied with spatulate setae; internal punctures sparse. Carina of ventral sensorial area of antennomeres elevated and visible in lateral view (Fig. 11), mainly after antennomere VIII; dorsal sensorial area of the antennomere XI (Fig. 10) elliptic, small, not reaching apex of antennomere; antennomere XI distinctly and abruptly sloped dorsally just after middle; antennomeres distinctly enlarged towards inferior edge (Fig. 10), more distinctly after antennomere IV.

Pronotum finely, sparsely punctate; punctures gradually becoming coarser and more abundant near lateral margin; lateral punctures with very small, spatulate seta. Prosternum coarsely, moderately densely punctate; nearly all punctures with small, spatulate seta. Prosternal process sparsely punctate (punctures with same type setae as prosternum). Mesosternum strongly, densely, deeply, confluently punctate, with a few very small, spatulated setae. Mesosternal process subglabrous and smooth. Mesepisterna coarsely, moderately densely punctate. Metasternum finely and sparsely punctate centrally; punctures gradually becoming coarser and denser towards lateral area; punctures close to metacoxae with small, spatulate setae. Elytra coarse; punctures dense on lateral parts and apical one-fourth; lateral punctures with very small thick setae, more conspicuously towards apices; punctures of apical one-third with small, spatulate setae; suture divergent at apex.

Urosternites densely punctate, mainly laterally; setae short, dense and spatulate, thick laterally; urosternite V with somewhat long, dense setae at margin, more conspicuous centrally (each seta apically spatulate). Coxae moderately sparsely punctate. Tibiae somewhat flat dorsally and sulcated laterally. Metatarsomere V (excluding claws) longer than I-III together.

**Dimensions in mm (**♀**)**. Total length (including mandibles), 13.4; prothorax: length, 2.5; anterior width, 2.9; posterior width, 2.7; humeral width, 3.4; elytral length, 7.9.

#### Type material.

 Holotype ♀ from INDONESIA, Sulawesi, *South East Sulawesi*: Konda (Kendari, Telkom Popalia; 1600 m; at light), 31.XII.2001, Akiko Saito col. (NSMT).

### Stenandra asiatica sp. n.

urn:lsid:zoobank.org:act:09AFC79D-E410-447C-B434-F78D31627F99

http://species-id.net/wiki/Stenandra_asiatica

Figs 12–14

Stenandra kolbei ; Santos-Silva et al. 2010: 73 (misidentification).

#### Etymology.

 The name refers to the continent where the species occurs.

#### Diagnosis.

 *Stenandra asiatica* sp. n. (Fig. 12) differs from *Stenandra vadoni* (Fig. 4) and *Stenandra kolbei* (Fig. 6) by the same characters of *Stenandra saitoae*sp. n. from Sulawesi, except the size of the dorsal sensorial area of the antennomere XI, which is large, and by the elytra not divergent apically (both similar to *Stenandra kolbei*). See diagnosis on *Stenandra saitoae* sp. n.

Female: Integument dark-brown; parts of mandibles, margins of pronotum, elytral suture, pro- and mesosternal process, trochanters, extreme ventral apices of tibiae blackish.

Dorsal face of head punctate coarsely for the most part; punctures sparser at central area, and coarse, dense and confluent near each side; punctures on area between upper eye lobes with short setae (lateral ones apically spatulate), more abundant near clypeus; punctures on area between posterior eye lobe and pronotum without setae. Area behind eyes coarsely punctate near the apices of upper eye lobes, sparsely near lower eye lobes; most punctures at the area close to upper eye lobe with a very small seta, slightly thicker at apex. Clypeus coarsely and densely punctate; punctures with short, spatulate setae. Labrum smooth and subglabrous except area near central projection with coarse punctures and short setae, in part spatulate, some only thick or acute apically. Upper eye lobe not notably distant from the base of antenna. Submentum coarse, moderately, densely punctate near anterior margin (confluently so centrally), gradually becoming sparse and coarse towards gena; each puncture with single, small seta (nearly all thicker apically; some of them spatulate; some acute apically); anterior margin slightly elevated throughout. Mentum with moderately sparse spatulate setae which somewhat longer laterally. Mandibles coarse; external punctures deep and dense, with small setae (very few spatulate); internal ones sparse. Carina of ventral sensorial area of antennomeres elevated and visible in lateral view (Fig. 14), mainly after antennomere VI; dorsal sensorial area of antennomere XI (Fig. 13) elliptic, large, almost reaching apex of antennomere; antennomere XI not abruptly sloped dorsally beyond middle; antennomeres distinctly enlarged towards inferior edge (Fig. 13), more distinctly after the antennomere IV.

Pronotum finely, sparsely punctate centrally, gradually becoming more coarsely and densely punctate laterally; lateral punctures with very small, spatulate seta. Prosternum coarsely, moderately, densely punctate, and nearly all punctures with small, apically spatulate seta. Prosternal process with small and spatulate setae. Mesosternum, densely, deeply, confluently punctate, and with small, spatulate setae. Mesepisterna coarsely, moderately, densely punctate. Metasternum finely and sparsely punctate centrally; punctures gradually becoming coarse, moderately abundant towards lateral area; lateral punctures close to metacoxae with small setae some of which spatulate. Elytra coarse; punctures dense on lateral parts and apical third; lateral punctures with very small seta, thicker towards apex and conspicuous from base to apex; nearly all punctures with small seta, more distinct towards apex (many spatulate, at least apically); suture not divergent at apex.

Urosternites densely punctate, mainly laterally; setae short, dense and spatulate (at least apically), mainly laterally; urosternite V with somewhat long, dense setae at margin, more conspicuous centrally (very few of them apically spatulate). Coxae moderately, sparsely punctate. Tibiae somewhat flat dorsally, and sulcate laterally. Metatarsomere V (excluding claws) longer than I-III together.

**Dimensions in mm (**♀**)**. Total length (including mandibles), 17.4; prothorax: length, 3.2; anterior width, 3.5; posterior width, 3.2; humeral width, 4.4; elytral length, 9.8.

#### Type material.

 Holotype ♀ from VIETNAM, *Vinh Phuc*: Tam Dao National Park, VII.14-27.1992, N. Katsura col. (MZSP).

## Supplementary Material

XML Treatment for Stenandra saitoae

XML Treatment for Stenandra asiatica
